# Possible Fossil Larvae of Staphylinidae from Kachin Amber and a Quantitative Morphological Comparison Indicate That Rove Beetle Larvae Partly Replaced Lacewing Larvae

**DOI:** 10.3390/insects16090910

**Published:** 2025-09-01

**Authors:** Joachim T. Haug, Ana Zippel, Gideon T. Haug, Carolin Haug

**Affiliations:** 1 Biocenter, Ludwig-Maximilians-Universität München, Großhaderner Str. 2, 82152 Planegg-Martinsried, Germany; joachim.haug@palaeo-evo-devo.info (J.T.H.); zippel@biologie.uni-muenchen.de (A.Z.); 2 GeoBio-Center at LMU, Richard-Wagner-Str. 10, 80333 Munich, Germany; 3 Fakultät für Biowissenschaften, Universität Heidelberg, Im Neuenheimer Feld 234, 69120 Heidelberg, Germany; gideon.haug@palaeo-evo-devo.info

**Keywords:** Staphylinidae, Burmese amber, Cretaceous, convergent evolution, ecological substitution

## Abstract

The highly diverse group of rove beetles, Staphylinidae, displays a great morphological variety across both adult and larval stages. However, due to the often cryptic habitats of their larvae, comprehensive descriptions of larval morphologies across extant ingroups remain incomplete. Also, the fossil record of rove beetle larvae appears to be sparse to date. We report here 35 newly identified fossil larvae of Staphylinidae from eight pieces of Myanmar Kachin amber, dating to the Cretaceous. Notably, one amber piece preserves three larval syninclusions, while another contains nine adults alongside twenty-six larvae, providing rare evidence of larval–adult co-occurrence in ancient environments. Given the predominantly predatory life cycle of extant rove beetles, the morphological similarities of these fossils to modern groups suggest comparable ecological roles in the Cretaceous, likely involving specialised forward-protruding mandibles adapted for a predatory lifestyle. A morphometric analysis of larvae of Staphylinidae alongside other extant and fossil predatory insect larvae still revealed significant differences between extant and fossil rove beetle larvae. Furthermore, Cretaceous rove beetle larvae differ from Cretaceous lacewing larvae in their head and mandible shape. In the modern fauna, the diversity of lacewing larvae is lower, and some modern rove beetle larvae now occupy areas formerly occupied by lacewing larvae. This result indicates that rove beetle larvae diversified after the Cretaceous, taking over certain ecological functions of lacewing larvae, likely representing a case of ecological substitution.

## Simple Summary

Beetles are highly diverse in their morphology, not only among adults, but also among the larvae. However, there is often only little knowledge about the larvae in particular. This also holds true for rove beetles, for which there is only scarce information about both extant and fossil larvae. We report here 35 new fossil rove beetle larvae, which are enclosed in eight pieces of Myanmar Kachin amber (ca. 100 million years old). Notably, one amber piece includes three larvae, while another piece contains nine adults alongside twenty-six larvae, which is a rare case of larvae and adults co-occurring in this ancient environment. As the fossil larvae look rather similar to modern rove beetle larvae, they probably also had a predatory lifestyle. Nevertheless, a quantitative analysis of the mouthpart shapes still revealed significant differences between extant and fossil rove beetle larvae and between fossil rove beetle larvae and co-occurring fossil lacewing larvae, which presumably were also predatory like their modern counterparts. In the modern fauna, the morphological diversity of lacewing larvae is lower, and the mouthparts of some modern rove beetle larvae look very similar to those of now extinct fossil lacewing larvae. This result indicates that rove beetle larvae became more diverse in the last 100 million years and took over certain ecological functions of lacewing larvae. This case likely represents an ecological substitution.

## 1. Introduction

Staphylinidae, the group of rove beetles, is extremely species-rich, with to date almost 67,000 formally described species in the extant fauna [1]. Over the years, it has become even more species-rich, as supposedly separate lineages were recognised as ingroups of Staphylinidae [2,3,4]. Rove beetles occur in various habitats, presenting a large ecological diversity [5,6,7,8] (see also [9] p. 396), and have an almost worldwide distribution (see [9] p. 394).

Molecular reconstructions (e.g., [10]) suggest that the lineage separated in the early Jurassic, almost 200 million years ago. With more than 400 fossil species [11], all based on adult specimens, rove beetles and their closer relatives (Staphylinoidea) also seem relatively well represented in the fossil record. Exceptionally detailed finds stem from amber deposits such as Baltic amber [12,13,14,15,16,17], but also from significantly older Cretaceous ambers [7,18,19,20,21,22,23,24,25,26,27,28,29,30,31,32,33,34,35,36,37,38,39,40,41,42]. The latter finds demonstrate that about 100 million years ago, a wealth of highly specialised morphologies and ecologies had already evolved in the rove beetle lineage.

As holometabolans, rove beetles spend a significant amount of time in their larval stages, as this is the phase of growth [43]. In fast-developing species, the entire larval phase may take only four weeks, while other species overwinter in the larval phase (see [44] p. 60). Rove beetle larvae are still understudied in many aspects, such as their morphology or life habits; while this statement is true for at least most insect larvae in general, the ratio of unknown larvae for formally described species within Staphylinidae seems unusually high (see [44] p. 55; [45] p. 343; [46] p. 317). Nevertheless some very detailed descriptions demonstrate that there is also quite a diversity of modern-day rove beetle larvae [3,47,48,49,50,51,52,53,54,55,56,57,58,59,60,61,62,63,64,65,66,67,68,69].

In the fossil record, larvae of rove beetles seem quite rare; there are only two specimens known: one from Dominican amber (see [70] fig. F-226 p. 136, refigured in [71] fig. 10. 30 p. 377; a staphylinoidean larva in [72] fig. E p. 88 seems not to be a rove beetle) and one from Kachin amber, Myanmar [73]. This scarcity of fossil rove beetle larvae is unexpected, given the rich fossil record of the adults and the fact that the animals spend a significant time in their larval phase, the latter increasing the time span during which potential enclosure in amber may occur. The scarcity may be an artefact, as, for example, rove beetle larvae in Baltic amber can be readily found on the market based on information from websites of traders, but have not been reported in scientific papers.

Here we report possible rove beetle larvae from Kachin amber, Myanmar. They differ from the already known one of Scydmaeninae from the same deposits [73] and expand the scarce record of these larvae. We furthermore perform a quantitative morphological analysis of the head and mandible shape in comparison to those of other predatory insect larvae. The results allow us to evaluate certain aspects of the evolution of ecological roles of rove beetle larvae over the last 100 million years.

## 2. Material and Methods

### 2.1. Material

Thirty-five fossil larvae preserved in eight amber pieces are at the centre of this study. There are 3 larvae of Staphylinidae within the amber piece PED 3744, and a total of 26 larvae of Staphylinidae within the amber piece PED 1302. All amber pieces originate from Kachin amber, Myanmar. Kachin amber has been suggested to be about 100 million years old (late Cretaceous) [74,75,76]. One amber piece with a single larva is part of the collection of the Nanjing Institute of Geology and Palaeontology of the Chinese Academy of Sciences (NIGPAS).

The other specimens were legally purchased on the trading platform ebay.com from the trader burmite-miner. The specimens have been deposited in the Palaeo-Evo-Devo Research Group Collection of Arthropods, Ludwig-Maximilians-Universität München (LMU Munich), Germany, under repository numbers PED 0067, 1302, 2115, 3368, 3744, 3982, and 4084. Fossils in Myanmar amber have caused discussion about ethics in palaeontological research [77,78]. Our strategy to improve the situation is to collaborate with colleagues in Myanmar [79,80].

### 2.2. Documentation and Preparation Methods

The specimen from NIGPAS was documented on a Discovery V16 stereo microscope (Zeiss, Oberkochen, Germany). Helicon Focus 7.0.2. stacking software (Helicon Soft, Kharkiv, Ukraine) was used to combine several images, overcoming limitations in depth of field. The other specimens were documented on a VHX-6000 digital microscope (Keyence, Osaka, Japan) from several available sides. Although the polishing removed most of the unevenness of the surface, a drop of glycerol was placed over the specimen and covered with a coverslip. A low-angle ring light was used for illumination. Images were recorded as composite images (fused stacks in z-axis, merged panoramas, HDR). Further processing (colour, contrast, etc.) was performed in Adobe Photoshop CS2 (Adobe, San José, CA, USA). Measurements of morphological characters were conducted using ImageJ 4.0.

Due to the partially unfortunate embedding position of the specimens within the amber pieces, the pieces were successively trimmed after documentation to access additional details. Rough grinding and cutting were performed with an electric hand-held rotary device (“Dremel”, but a different brand, FCT-300 Combitool, Ferm, Zwolle, The Netherlands). Further grinding was performed with abrasive paper of grain sizes 600 and 1200. Final polishing was performed with silver polish (Poliboy).

### 2.3. Description Style for New Specimens

In the Results section, we use a straightforward descriptive style to make the identification of structures in the new specimens clear to both specialists and non-specialists. Where relevant, we include terms commonly used by beetle specialists for larvae of Staphylinidae in brackets.

### 2.4. Shape Analysis

In order to compare fossil and extant larvae with each other and in a broader frame, we compared the head shapes including the mandibles of extant larvae of Staphylinidae, of some of the fossils reported here (those in which the head was available in the right orientation) and of other larvae that have some general resemblance to these. For the latter, we included lacewing larvae with their prominent mandible–maxilla compounds, using those in which these mouthparts were simple sickle-shaped (extant larvae of Hemerobiidae, Chrysopidae, Psychopsidae, some of Crocinae and their fossil relatives), as well as larvae of diving beetles (Dytiscidae). Data for these groups were retrieved from the literature:

- Silky lacewings (Psychopsidae) [81,82,83,84,85,86,87,88,89,90,91,92,93];
- Thread-winged lacewings (Crocinae) and spoon-winged lacewings (Nemopterinae) [94,95,96,97,98,99,100,101,102,103,104,105,106,107,108,109,110,111,112,113,114,115];
- Brown lacewings (Hemerobiidae) [116,117,118,119,120,121,122,123,124,125,126,127,128,129];
- Green lacewings (Chrysopidae) [130,131,132,133,134,135,136,137,138,139,140,141,142,143,144,145,146,147,148,149,150,151,152,153,154,155];
- Fossil aphidlions (Hemerobiidae, Chrysopidae, or unclear) [70,71,80,156,157,158,159,160,161,162,163,164,165,166,167,168,169];
- Publications with different groups of Neuroptera [170,171,172,173,174,175,176,177,178,179,180,181];
- Diving beetles (Dytiscidae) [182,183,184,185,186,187,188,189,190,191,192,193,194,195,196,197,198];
- Rove beetles (Staphylinidae) [3,4,55,60,63,66,67,68].
- In addition to publications, some specimens were retrieved from image repositories such as bugguide.com (see Supplementary Table S1).

Three analyses were performed. The first analysis included 597 specimens (Supplementary Files S01–S07). The analysis proved strongly polarised due to some aberrant morphologies of certain diving beetle larvae, which obscured differences in the larger set of the specimens. We therefore analysed this subset in a second analysis with 580 specimens separately (Supplementary Files S08–S14). In a third analysis, we analysed only extant larvae of Staphylinidae and three of the fossils (in total 34 specimens; Supplementary Files S15–S21).

## 3. Results

### 3.1. General Description of Body Organisation of the Larvae

Body campodeiform and organised in anterior head and posterior trunk. Head composed of six segments: ocular segment and post-ocular segments 1–5. Ocular segment often represented anteriorly by nasale (frons continuous with clypeus and labrum), but sometimes labrum separated from clypeus by distinct furrow. Post-ocular segments 1 and 3–5 with appendages (antennae, mandibles, maxillae, labium), post-ocular segment 2 (intercalary segment) without appendages. The feeding appendages facing forwards (prognathous). Each maxilla with cardo, stipes, endite (probably conjoined lacinia and galea, so-called mala) and maxillary palp. Trunk subdivided into anterior thorax and posterior abdomen. Thorax with three segments and abdomen with nine discernible units (eight segments and trunk end with pygopod). Pygopod sometimes with membranous lobes posteriorly. Thorax segments each with a pair of locomotory appendages (legs). Each leg with five elements: coxa, trochanter, femur, tibia, and tarsungulum. No discernible locomotory appendages present on abdomen, trunk end with paired urogomphi. Each urogomphus either as a single unit bearing a seta at its distal tip, or each urogomphus subdivided into two parts (so-called ‘articulated urogomphi’), with the distal part bearing a seta at its distal tip.

### 3.2. Description of Specimen PED 3982

**Figure 1:**
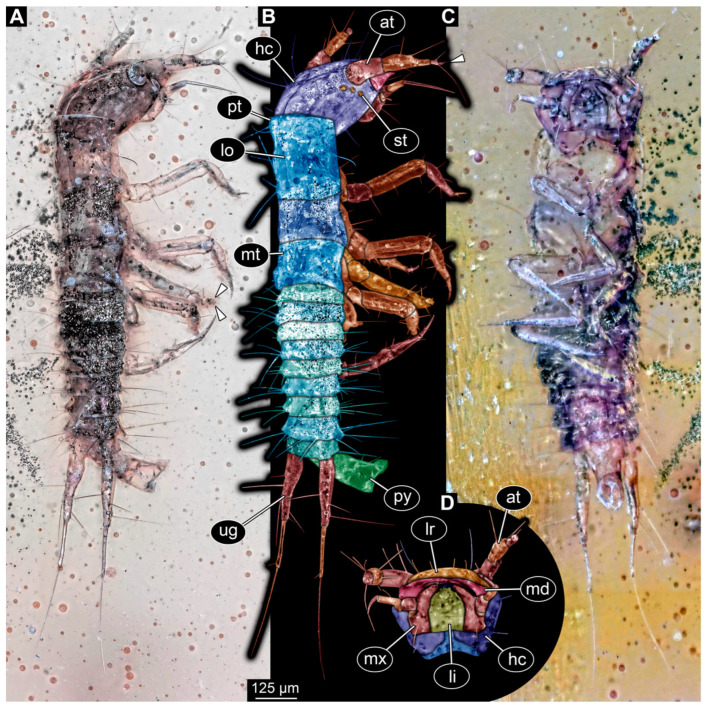
Fossil specimen PED 3982, larva of Staphylinidae. (**A**) Habitus in dorso-lateral view, arrows mark two small setae on tarsungulum. (**B**) Colour-marked version of **A**, arrow marks set of small sensory hairs on the distal element of antenna. (**C**) Habitus in ventral view. (**D**) Colour-marked version of head from **C**. Abbreviations: at = antenna; hc = head capsule; li = labium; lo = longitudinal moulting suture; lr = labrum; md = mandible; mt = metathorax; mx = maxilla; pt = prothorax; py = pygopod; st = stemma; ug = urogomphus.

Specimen accessible in ventral and dorso-lateral view (Figure 1). Small particles of syninclusions present in amber piece, partially covering the specimen. Slight Verlumung present around specimen. Total body length ~1.32 mm.

Head: Head capsule hexagonal in ventral view (Figure 1C), at widest point wider than medially long, ~1.3× (~0.24 mm long). Dorsal moulting line (ecdysial suture) discernible, with long median suture, with two arms (V-shape) (Figure 1A,B). Anterior part of head capsule with at least four setae of same length and two longer ones (Figure 1C,D). No anterior teeth-like structures discernible on anterior part of head capsule in ventral view (Figure 1C,D). Three stemmata discernible laterally on head capsule (Figure 1B), possibly additional stemmata present. Antennae with three elements (antennomeres) discernible, possibly proximal element subdivided into two, head capsule longer than antennae, 1.4× (antennae ~0.17 mm long). All elements longer than wide, penultimate element with an oblique distal margin with no sensory process discernible (possibly due to preservation and angle). Penultimate element distally with two setae, a shorter and a longer one (Figure 1B). Ultimate element with short sensory setae distally (solenidia; Figure 1B).

Mandibles sickle-shaped, ~0.2 mm long from functionally anterior tip to the head capsule in ventral view. Maxilla (not including palp) longer than wide proximally, 2.8× (~0.15 mm long). Each stipes with one simple seta proximally. Maxillary palp with four elements (palpomeres), ~0.13 mm long (Figure 1D). Labium longer than wide (~0.12 mm long), labial palps and ligula sclerite discernible (Figure 1D).

Trunk: Prothorax ~0.25 mm long, anterior part of tergite covering posterior part of head capsule, longest segment of thorax in lateral view (Figure 1A,B). Longitudinal moulting suture discernible medially (Figure 1B). Meso- and metathorax similar, wider than long in ventral view, both ~0.13 mm long (Figure 1A–C). Legs almost as long as thorax, thorax 1.1× longer (legs ~0.45 mm long). Coxa of prothorax leg with at least two setae, trochanter with two longer simple setae, femur longest element of leg with several simple setae of similar length, tibia with at least four simple setae, and tarsungulum with two small spines (Figure 1A: arrows).

Abdomen ~0.52 mm long, abdomen units 1–9 wider than long (between 0.05 and 0.07 mm long and between 0.19 and 0.15 mm wide), width of units slightly tapering posteriorly (Figure 1A–C). Each unit laterally on both sides with 2–3 longer setae and some units with additional shorter setae. Trunk end with two urogomphi posteriorly (urogomphi ~0.66 mm long). Urogomphi with two parts: proximal part wider with several moderately long and long setae, and distal part thinner with a single short seta laterally and one very long seta distally (Figure 1A–C). Pygopod elongated cylindric in both ventral and dorso-lateral view, with wider posterior part with lobes (trunk end: ~0.2 mm long) (Figure 1A–C).

### 3.3. Description of Specimen PED 0067

**Figure 2:**
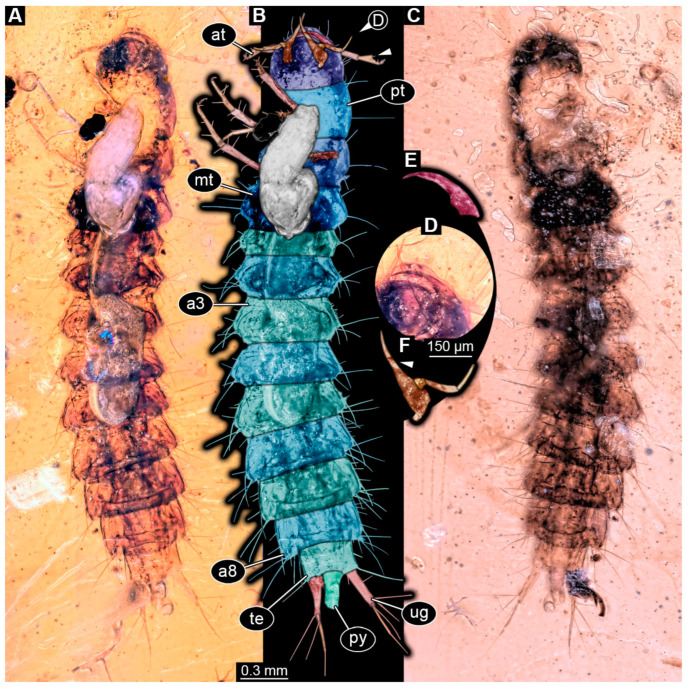
Fossil specimen PED 0067, larva of Staphylinidae. (**A**) Habitus in ventral view. (**B**) Colour-marked version of **A**, arrow marks seta-like sensory process of the penultimate element of antenna. (**C**) Habitus in dorsal view. (**D**) Partial close-up of head in ventral view. (**E**) Colour-marked mandible from **D**. (**F**) Colour-marked maxilla from **D**, arrow marks long endite (mala?). Abbreviations: a3–8 = abdomen segments 3–8; at = antenna; mt = metathorax; pt = prothorax; py = pygopod; te = trunk end; ug = urogomphus.

Specimen accessible in ventral and dorsal view (Figure 2A–C). Small particles of syninclusions and bubbles present in amber piece, partially covering the specimen. Specimen partially damaged. Total body length ~3.64 mm.

Head: Head capsule semicircular in shape in ventral view (Figure 2B), at widest point wider than medially long, ~1.1× (~0.4 mm long). Dorsal moulting line (ecdysial suture) not discernible due to preservation of specimen (Figure 2C). Anterior part of head capsule (presumed labrum) with two simple, short setae and two longer ones laterally from the short ones. No anterior teeth-like structures discernible on anterior part of head capsule in ventral view (Figure 2A,B). Stemmata not discernible but presumed. Antennae with three elements (antennomeres) discernible, head capsule longer than antennae, 1.2× (antennae ~0.34 mm long). All elements longer than wide, penultimate element oblique with a seta-like sensory process discernible medially (Figure 2B). Ultimate element distally with two long setae and at least one short sensory seta (solenidium).

Mandibles sickle-shaped, ~0.3 mm long from functionally anterior tip to the head capsule in ventral view (Figure 2D,E). Maxilla (not including palp) longer than wide proximally, 3.5× (~0.34 mm long). Endite (mala?) elongate, ~0.21 mm long, reaching outwards beyond anterior rim of head capsule (Figure 2D,F). Maxillary palp with three elements (palpomeres), ~0.25 mm long. Labium not discernible due to preservation but presumed.

Trunk: Prothorax ~0.31 mm long, longest segment of thorax in ventral view (Figure 2B). Longitudinal moulting suture not discernible due to preservation. Meso- and metathorax similar, wider than long in ventral view, both ~0.28 mm long. Legs relatively thin, total length not measurable due to preservation (Figure 2A,B). Femur longest element of leg, at least 0.31 mm long with several simple setae of similar length, tibia ~0.3 mm long with at least four simple setae, and tarsungulum ~0.07 mm long.

Abdomen ~2.36 mm long, abdomen units 1–9 wider than long (between 0.14 and 0.27 mm long and between 0.31 and 0.69 mm wide), segment 5 widest, trunk end narrowest. Each unit laterally on both sides with 2–3 longer setae and several shorter setae. Trunk end with two urogomphi posteriorly (urogomphi ~0.62 mm long). Urogomphi with two parts: proximal part wider with several short setae laterally and three long setae posteriorly, and distal part thinner with moderately long seta distally (Figure 2A–C). Pygopod elongated, cylindric in both ventral and dorso-lateral view, shorter than proximal part of urogomphi (trunk end ~0.23 mm long; Figure 2A–C).

### 3.4. Description of Specimen PED 2115

**Figure 3:**
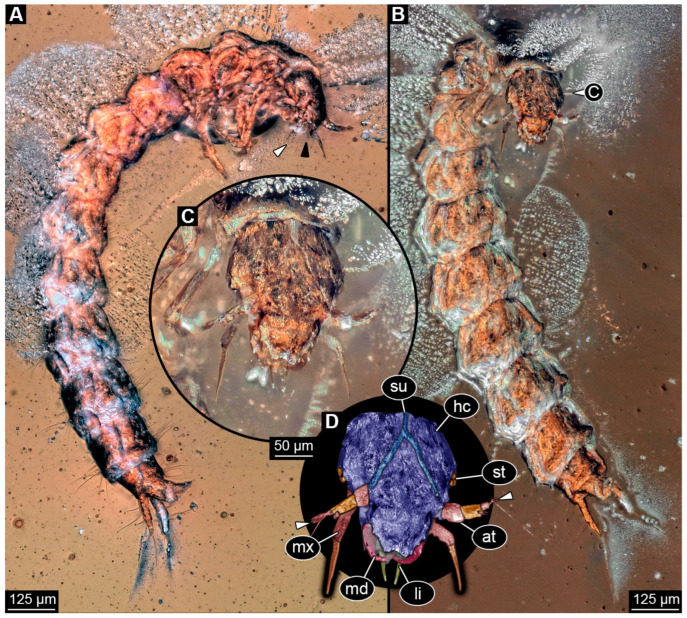
Fossil specimen PED 2115, larva of Staphylinidae. (**A**) Habitus in lateral view, white arrow marks labial palps, black arrow marks possible endite of maxilla. (**B**) Habitus in ventral view. (**C**) Close-up of the head from (**B**). (**D**) Colour-marked version of **C**, arrows mark set of small sensory hairs on the ultimate element of antenna. Abbreviations: at = antenna; hc = head capsule; li = labium; md = mandible; mx = maxilla; st = stemma; su = ecdysial suture.

Specimen accessible in ventral and lateral view (Figure 3A,B). Small particles of syninclusions and bubbles present in amber piece, partially covering the specimen. Slight Verlumung present around specimen. Total body length ~1.77 mm.

Head: Head capsule elongated in dorsal view but widening between lateral areas with stemmata, rather pear-shaped (Figure 3C,D), longer than wide at widest point, ~1.2× (~0.18 mm long). Dorsal moulting line (ecdysial suture) discernible, with short median suture, with two arms (V-shape) (Figure 3C,D). Anterior part of head capsule forming nasale, nasale anteriorly with median notch surrounded by two larger anterior teeth-like structures and several smaller lateral ones (Figure 3C,D). Three stemmata discernible laterally on each side of head capsule (Figure 3C,D), probable additional stemmata present, likely five per side. Antennae with three elements (antennomeres) discernible, head capsule longer than antennae, 1.8× (antennae ~0.1 mm long). Proximal element almost as wide as long, penultimate element longer than wide, oblique in dorsal view with thin sensory process, ultimate element oval in dorsal view. Ultimate element laterally with at least one longer seta at the middle and distally with short sensory setae (solenidia; Figure 3D).

Mandibles partially discernible, sickle-shaped, possibly with a tooth, ~0.06 mm long from functionally anterior tip to the head capsule in dorsal view (Figure 3D). Maxilla partially obscured, maxillary palp with three elements (palpomeres) discernible. Labium partially obscured, labial palps reaching outwards beyond anterior rim of head capsule.

Trunk: Prothorax ~0.15 mm long, longest segment of thorax in lateral view (Figure 3A). Longitudinal moulting suture not discernible due to preservation. Meso- and metathorax ~0.14 mm long. Legs relatively thin, thorax longer than legs, 1.3× (legs ~0.32 mm long; Figure 3A,B). Coxa ~0.02 mm long, trochanter ~0.09 mm long, femur longest element of leg, ~0.11 mm long, tibia ~0.07 mm long, and tarsungulum ~0.03 mm long.

Abdomen ~1.27 mm long, abdomen segments 1–2 partially obscured in ventral view, abdomen units 3–9 wider than long in ventral view (between 0.09 and 0.16 mm long and between 0.11 and 0.2 mm wide), segment 6 widest, trunk end narrowest (Figure 3A,B). Each unit laterally on both sides with several setae. Trunk end with two urogomphi posteriorly (urogomphi ~0.12 mm long). Urogomphi as a single part, with several short setae and one longer seta laterally and a single long seta distally (Figure 3A,B). Pygopod cylindric in both ventral and lateral view, urogomphi longer than trunk end, 1.5× (trunk end ~0.08 mm long) (Figure 3A,B).

### 3.5. Description of Specimen PED 3368

**Figure 4:**
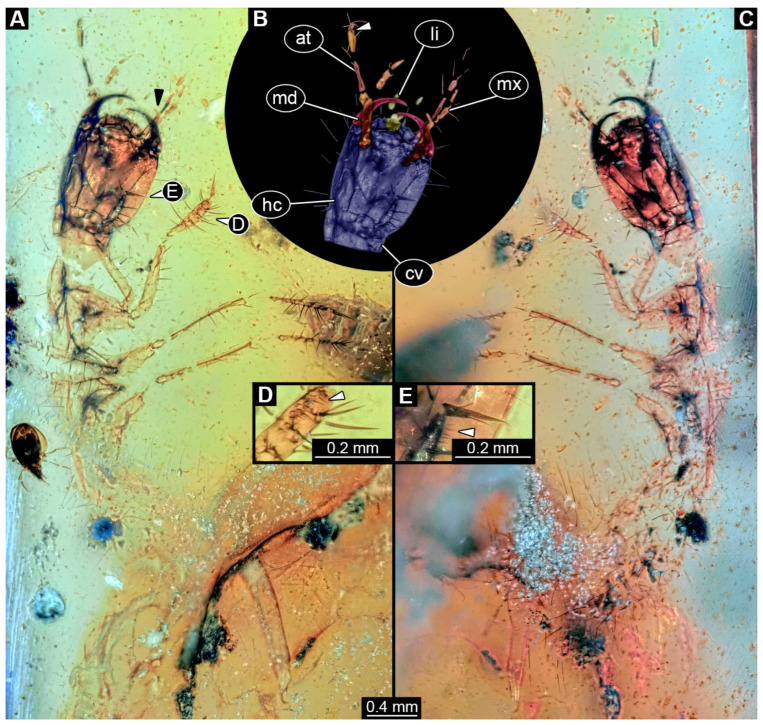
Fossil specimen PED 3368, larva of Staphylinidae. (**A**) Habitus in ventro-lateral view, partially torn apart and obscured by syninclusions. (**B**) Colour-marked head from (**A**), small arrow marks sensory process on penultimate element of antenna. (**C**) Habitus in dorso-lateral view. (**D**) Close-up of tibia of prothorax leg, arrow marks minute setae arranged in a comb. (**E**) Close-up of tarsungulum with two small and a single minute seta, arrow marks setal comb on tibia of leg of prothorax. Abbreviations: at = antenna; cv = cervix; hc = head capsule; li = labium; md = mandible; mx = maxilla.

Specimen accessible in ventral and dorsal view (Figure 4A,C). Small particles of syninclusions (including a single biosyninclusion of a mite) and bubbles present in amber piece, partially covering the specimen. Specimen partially damaged and broken into several parts. Total body length not available due to preservation and incompleteness of specimen.

Head: Head capsule oval in dorsal view (Figure 4B), longer than wide at widest point, ~1.3× (~0.95 mm long), with neck region posteriorly (cervix: ~0.13 mm long). Dorsal moulting line (ecdysial suture) discernible, with short median suture, with two arms (V-shape) (Figure 4C). Anterior part of head capsule forming nasale, nasale anteriorly with four anterior teeth-like structures (Figure 4A). No stemmata discernible, but probable stemmata presumed. Antennae broken into pieces and partially obscured, but four elements (antennomeres) discernible, total length not available. All elements longer than wide, penultimate element oblique with small sensory process discernible medially (Figure 4A,B: arrow). Ultimate element distally with three long setae and at least one short sensory seta (solenidium, Figure 4A,B).

Mandibles sickle-shaped, ~0.48 mm long from functionally anterior tip to the head capsule in dorsal view (Figure 4A–C). Maxilla elongated: cardo and stipes elongated, endite (mala?) finger-like (Figure 4A: black arrow), maxillary palp broken into pieces, number of elements (palpomeres) not discernible. Labium relatively short, labial palps with multiple elements, ligular sclerite (ligula) discernible. Labial palps and ligula reaching outwards beyond anterior rim of head capsule (Figure 4B).

Trunk: All trunk segments and trunk end either distorted or not accessible due to damage and preservation of specimen. Legs relatively thin and long (legs ~1.78 mm long; Figure 4A,C). Coxa ~0.35 mm long, trochanter ~0.24 mm long with several spine-like setae, femur longest element of leg, ~0.54 mm long with multiple spine-like setae, tibia ~0.44 mm long with multiple longer and tiny spine-like setae in formation of tibial comb (Figure 4D, E: arrow), and tarsungulum ~0.21 mm long with two short setae and one minute seta (Figure 4E).

Femur longest element of leg (at least 0.31 mm long) with several simple setae of similar length, tibia (~0.3 mm long) with at least four simple setae and tarsungulum (~0.07 mm long).

Abdomen units only partially discernible, heavily setose. Urogomphi not discernible. Pygopod not discernible.

### 3.6. Description of Specimens in PED 3744

**Figure 5:**
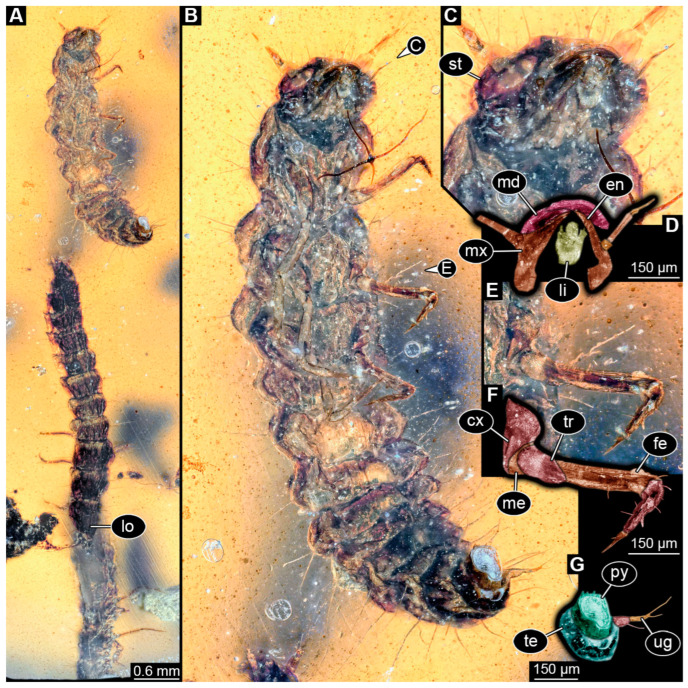
Three fossil specimens in amber piece PED 3744, larvae of Staphylinidae. (**A**) Habitus of specimen 1 in ventral view, specimen 2 in dorsal view and specimen 3 in lateral view. (**B**) Close-up of specimen 1 in ventral view. (**C**) Close-up of head from (**B**). (**D**) Colour-marked mouthparts of (**C**). (**E**) Close-up of mesothorax leg from (**B**). (**F**) Colour-marked version of (**E**). (**G**) Colour-marked version of trunk end with pygopod and urogomphus from (**B**). Abbreviations: cx = coxa; en = endite; fe = femur; li = labium; lo = longitudinal moulting suture; md = mandible; me = membrane; mx = maxilla; py = pygopod; st = stemma; te = trunk end; tr = trochanter; ug = urogomphus.

**Figure 6:**
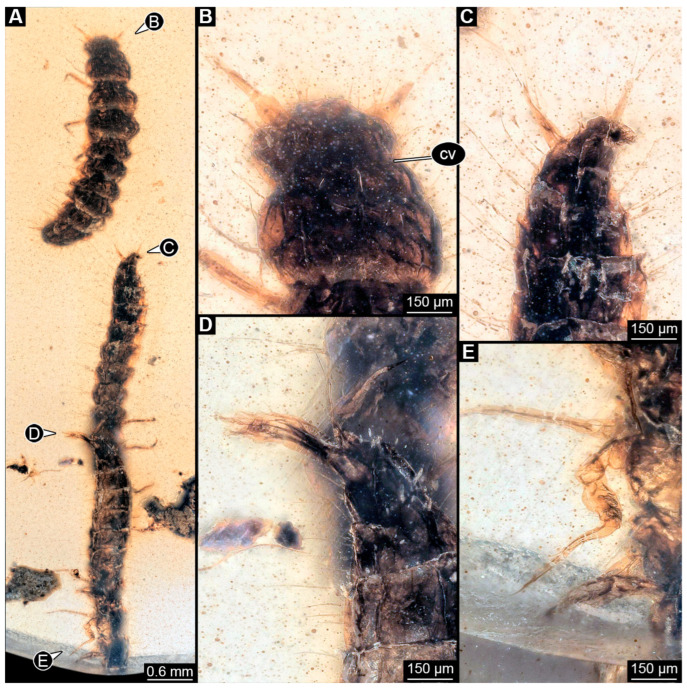
Three fossil specimens in amber piece PED 3744, larvae of Staphylinidae, continued. (**A**) Habitus of specimen 1 in dorsal view, specimen 2 in ventral view and specimen 3 in dorso-lateral view. (**B**) Close-up of head from specimen 1 from (**A**). (**C**) Close-up of trunk end with urogomphi and pygopod from specimen 2 from (**A**). (**D**) Close-up of trunk end with urogomphi and pygopod from specimen 3 from (**A**). (**E**) Close-up of legs from specimen 3 from (**A**). Abbreviation: cv = cervix.

The amber piece contains three biosyninclusions. All larvae appear similar and are described together as a single morphotype. Two specimens are accessible in ventral and dorsal view, one in lateral view (Figure 5 and Figure 6). Small particles of syninclusions and bubbles are present in the amber piece and are partially obscuring the specimens (e.g., stellate hairs of plants).

Head: Head capsule oval in dorsal view (Figure 6B), wider than long, ~1.8× (~0.19 mm long), with neck region (cervix) posteriorly (~0.13 mm long). Dorsal moulting line (ecdysial suture) not discernible due to preservation of specimens (Figure 5A and Figure 6B). Anterior part of head capsule (presumed labrum) with several simple short setae and several longer ones. No anterior teeth-like structures discernible on anterior part of head capsule in ventral view (Figure 5C). A single stemma discernible laterally on side of head capsule (Figure 5C), probable additional stemmata present. Antennae with three or four elements (antennomeres) discernible, antennae as long as head capsule (antennae ~0.19 mm long). Penultimate element oblique with no sensory process discernible (possibly due to preservation and angle; Figure 5B). Ultimate element distally with several long setae (Figure 6B).

Mandibles sickle-shaped, ~0.22 mm long from functionally anterior tip to the head capsule in ventral view (Figure 5C,D). Maxilla (not including palp) longer than wide proximally (~0.28 mm long). Maxilla elongated, cardo and stipes together elongated, endite (mala?) prominent and long (Figure 5D), maxillary palp with three elements (palpomeres), ~0.22 mm long. Labium longer than wide, labial palps with two elements (palpomeres) and broad ligula sclerite discernible.

Trunk: Prothorax ~0.34 mm long, longest segment of thorax in dorsal view (Figure 6A,B). Possible longitudinal moulting suture discernible on specimen 2 in dorsal view (Figure 5A). Mesothorax ~0.31 mm long and metathorax ~0.22 mm long. Legs relatively thin, legs as long as thorax (legs ~0.87 mm long) (Figure 5E,F and Figure 6E). Coxa ~0.12 mm long, trochanter ~0.16 mm long, femur longest element of leg, ~0.31 mm long with several simple setae of similar length, tibia ~0.18 mm long with multiple shorter setae, and tarsungulum ~0.09 mm long with single short seta.

Abdomen ~2.2 mm long, abdomen units 1–9 wider than long in ventral view (between 0.08 and 0.17 mm long and between 0.16 and 0.5 mm wide), segment 4 widest, trunk end narrowest (Figure 5A and Figure 6A). Each unit laterally on both sides with several setae. Trunk end with two urogomphi posteriorly (urogomphi ~0.23 mm long). Urogomphi possibly with two parts: proximal part wider, distal part thinner with moderately long seta posteriorly at functionally posterior tip (Figure 6D). Pygopod elongated, cylindric in dorso-lateral view, longer than urogomphi with posterior part with lobes (trunk end between 0.24 and 0.44 mm long) (Figure 5B,G and Figure 6C,D).

### 3.7. Description of the Specimen from NIGPAS

**Figure 7:**
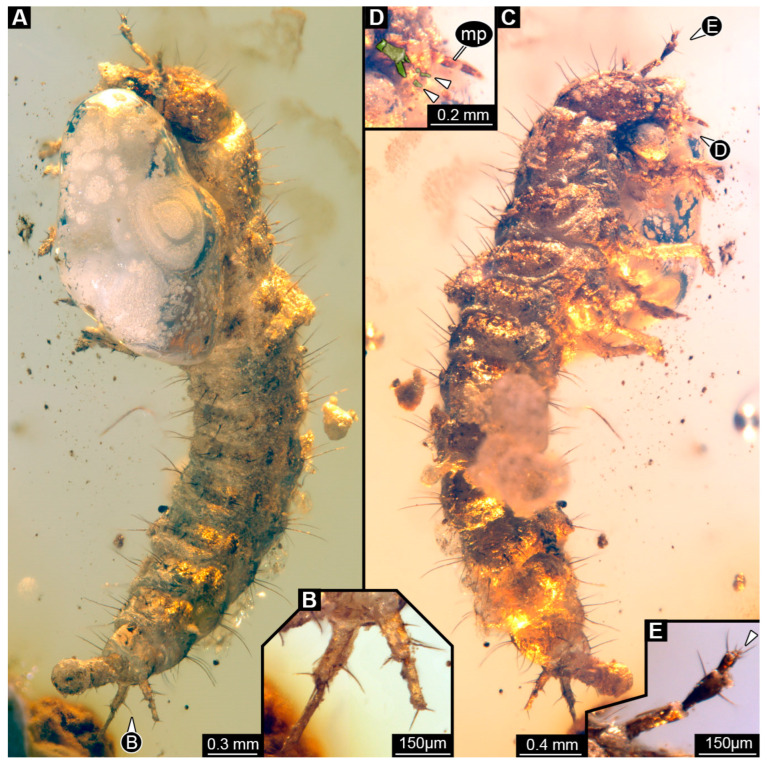
Fossil specimen from NIGPAS, larva of Staphylinidae. (**A**) Habitus of specimen in lateral view. (**B**) Close-up of urogomphi from (**A**). (**C**) Habitus of specimen in lateral view, other side than **A**. (**D**) Close-up of mouthparts from (**C**) with colour-marked labium; arrows mark palps of labium. (**E**) Close-up of antenna from (**C**), arrow marks set of sensory hairs on the ultimate element of antenna. Abbreviations: mp = maxillary palp.

Specimen accessible in both lateral views (Figure 7A,C). Small particles of syninclusions and large bubbles present in amber piece, partially covering the specimen. Total body length ~4.98 mm.

Head: Head capsule ~0.71 mm long. Dorsal moulting line (ecdysial suture) discernible, but shape of two arms not discernible (Figure 7C). Anterior part of head capsule protruding forward anteriorly, shape not discernible (Figure 7A). No stemmata discernible, but probable stemmata presumed. Antennae with four elements (antennomeres) discernible, however proximal part partially covered by Verlumung, head capsule longer than antennae, 1.8× (antennae ~0.39 mm long; Figure 7E). All elements longer than wide, penultimate element oblique with sensory process discernible. Penultimate element with two longer setae. Ultimate element with two longer setae and distally with four short sensory setae (solenidia; Figure 7E: arrow).

Mandibles not discernible due to preservation. Maxilla discernible only by maxillary palp with three accessible elements (palpomeres), ~0.21 mm long (Figure 7D). Labium only partially discernible due to bubble, with palps with at least two elements (Figure 7D), ligula small, triangular in ventro-lateral view, narrowing distally (Figure 7D).

Trunk: Prothorax ~0.57 mm long, longest segment of thorax in lateral view (Figure 7A,C). Mesothorax ~0.37 mm long and metathorax ~0.28 mm long. Legs relatively thin, thorax longer than legs, 1.4× (legs ~0.87 mm long) (Figure 7A,C). Coxa ~0.11 mm long, trochanter ~0.23 mm long with multiple simple spine-like setae of similar length, femur longest element of leg, ~0.35 mm long with several simple spine-like setae of similar length, tibia ~0.21 mm long with multiple simple spine-like setae of different lengths, and tarsungulum ~0.06 mm long with no discernible setae.

Abdomen ~2.2 mm long, abdomen units 1–9 wider than long in ventro-lateral view (between 0.18 and 0.4 mm long and between 0.35 and 0.77 mm wide), segment 4 widest, trunk end narrowest (Figure 7A,C). Each unit laterally on both sides with several setae. Trunk end with two urogomphi posteriorly (urogomphi ~0.33 mm long). Urogomphi with two parts: proximal part wider with several short setae and several moderately long setae laterally, and distal part thinner with two short setae distally (Figure 7B). Pygopod elongated, cylindric with posterior part with lobes in lateral view, slightly longer than urogomphi, 1.1× (trunk end ~0.36 mm long) (Figure 7A,C).

### 3.8. Description of PED 1302

**Figure 8:**
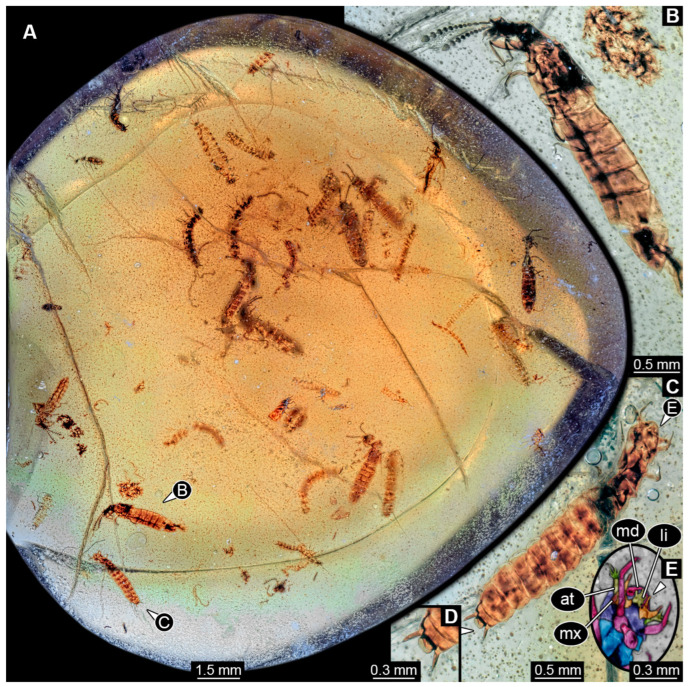
Several dozens of fossil specimens in amber piece PED 1302, adults and larvae of Staphylinidae. (**A**) Overview of amber piece with all specimens. (**B**) Habitus of an adult specimen in lateral view. (**C**) Habitus of a larva in ventral view. (**D**) Close-up of trunk end with urogomphi and pygopod from (**C**). (**E**) Colour-marked head with partial prothorax and legs from (**C**), arrow marks endite (mala?). Abbreviations: at = antenna; li = labium; md = mandible; mx = maxilla.

**Figure 9:**
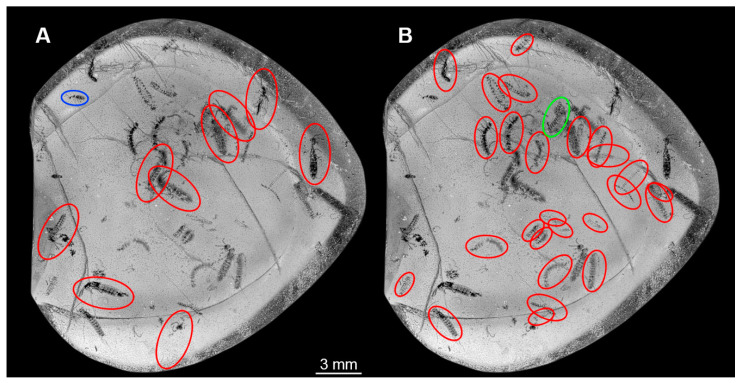
Several dozens of fossil specimens in amber piece PED 1302, adults and larvae of Staphylinidae, continued, plus unidentified adult and larva of Coleoptera. (**A**) Grey-scale image of amber piece, adults of Staphylinidae circled in red colour, an unidentified adult beetle circled in blue colour. (**B**) Grey-scale image of amber piece, larvae of Staphylinidae circled with red colour, unidentified larva circled with green colour.

The amber piece contains numerous biosyninclusions (Figure 8 and Figure 9). Nine of these can be identified as adults of the group Staphylinidae (Figure 8A,B and Figure 9A). A total of 26 specimens appear to be larval forms (Figure 9B); some show short urogomphi and a protruding anal membrane (Figure 8C,D). One specimen is of unclear state (Figure 9B). In addition, a small adult beetle is preserved (Figure 9A). The preservation of the specimen is not very good; many details are not accessible. Also, the amber is full of debris. Grinding closer to the specimens could in theory improve the resolution of details, yet with so many syninclusions it could not be executed without destroying some of the latter. As the piece contains information about co-occurrence, we hesitated to perform any grinding.

Head of larvae: Head capsule semicircular in shape in dorsal and ventral view (Figure 8A,C), wider than long, 1.2× (~0.31 mm long), possibly with neck region (cervix) posteriorly. Dorsal moulting line (ecdysial suture) not discernible due to preservation (Figure 8A). Anterior part of head capsule (presumed labrum) not discernible due to preservation (Figure 8E). No anterior teeth-like structures discernible on anterior part of head capsule in ventral view (Figure 8E). No stemmata discernible, but probable stemmata presumed. Antennae with multiple elements (antennomeres) discernible, head capsule longer than antennae, 1.9× (antennae ~0.16 mm long; Figure 8E). Ultimate element with two longer setae and distally with four short sensory setae (solenidia; Figure 8E).

Mandibles ~0.11 mm long from functionally anterior tip to the head capsule in ventral view (Figure 8A,C). Maxilla partially obscured, possible mala narrow and long (Figure 8E: arrow), maxillary palp with multiple elements (palpomeres) discernible, ~0.18 mm long. Labium longer than wide (~0.11 mm long), labial palps discernible, ligula sclerite tapering distally (Figure 8C).

Trunk of larvae: Prothorax ~0.24 mm long, longest segment of thorax in dorsal view (Figure 8A). Mesothorax ~0.22 mm long and metathorax ~0.18 mm long. Legs relatively thin, thorax longer than legs, 1.3× (legs ~0.51 mm long) (Figure 8A,C). Differentiation of coxa and trochanter not discernible, femur ~0.17 mm long, tibia ~0.14 mm long and tarsungulum ~0.03 mm long with two spines laterally opposite to each other.

Abdomen ~2.1 mm long, abdomen units 1–9 wider than long in dorsal view (between 0.12 and 0.3 mm long and between 0.29 and 0.55 mm wide), segment 6 widest, trunk end narrowest (Figure 8A,C). Each unit laterally on both sides with several setae. Trunk end with two urogomphi posteriorly (urogomphi ~0.16 mm long). Urogomphi with a single part, with several short setae laterally and a single short seta distally (Figure 8A,C). Pygopod cylindric in lateral view, slightly wider than long with two simple setae posteriorly, urogomphi longer than trunk end, 1.6× (trunk end ~0.1 mm long) (Figure 8A,C).

### 3.9. Description of Specimen PED 4084

**Figure 10:**
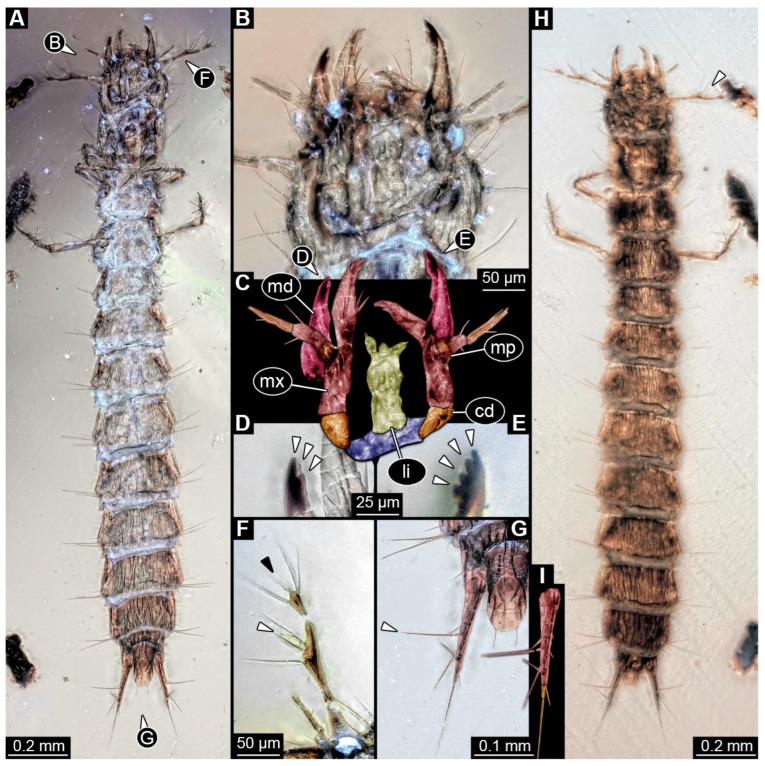
Fossil specimen PED 4084, larva of Staphylinidae: (**A**) Habitus of larva in ventral view. (**B**) Close-up of head from (**A**). (**C**) Colour-marked mouthparts of (**B**). (**D**) Close-up of tip of right mandible, arrows mark teeth. (**E**) Close-up of left mandible, arrows mark teeth. (**F**) Close-up of antenna from (**A**), white arrow marks cone-shaped sensory process on penultimate element of antenna, black arrow marks set of short sensory setae on the ultimate element of antenna. (**G**) Close-up of pygopod and urogomphus from (**A**), arrow marks fringed seta. (**H**) Habitus of larva in dorsal view, arrow marks cone-shaped sensory process on penultimate element of antenna. (**I**) Colour-marked version of urogomphus from (**G**). Abbreviations: cd = cardo; li = labium; md = mandible; mp = maxillary palp; mx = maxilla.

Specimen accessible in ventral and dorsal view (Figure 10A,H). Particles of syninclusions and small bubbles present in amber piece, partially covering the specimen. Total body length ~2.16 mm.

Head: Head capsule circular in shape in dorsal view (Figure 10H), as wide as long (~0.23 mm long). Dorsal moulting line (ecdysial suture) not discernible due to preservation (Figure 10H). Anterior part of head capsule (presumed labrum) with at least three setae per side anteriorly (Figure 10H). No anterior teeth-like structures discernible on anterior part of head capsule in ventral view (Figure 10A). No stemmata discernible, but probable stemmata presumed. Antennae with three elements (antennomeres) discernible, head capsule longer than antennae, 1.2× (antennae ~0.19 mm long), penultimate element longer than proximal element (Figure 10F). All elements longer than wide, penultimate element oblique with relatively big cone-shaped sensory process discernible (Figure 10F,H: white arrow). Penultimate element with three longer setae. Ultimate element with four longer setae and distally with at least four short sensory setae (solenidia; Figure 10F).

Mandibles asymmetric, ~0.13 mm long from functionally anterior tip to the head capsule in ventral view (Figure 10B,C). One mandible with three teeth, one with four teeth distally (Figure 10D,E). Proximal part of maxilla (not including palp) longer than wide proximally (~0.19 mm long), cardo triangular in ventral view, stipes elongated, endite (mala?) large and long with fringed tip medially (Figure 10C), maxillary palp with three elements (palpomeres), ~0.1 mm long. Penultimate element longer than proximal element. Labium longer than wide (~0.11 mm long), labial palps with two elements (palpomeres) and broad ligula sclerite (ligula) discernible.

Trunk: Prothorax ~0.18 mm long, longest segment of thorax in dorsal view (Figure 10A,H). Mesothorax and metathorax subsimilar, ~0.16 mm long. Legs relatively thin, thorax longer than legs, 1.9× (legs ~0.34 mm long) (Figure 10A,H). Coxa ~0.03 mm long, trochanter ~0.04 mm long with multiple simple setae of similar length, femur longest element of leg, ~0.13 mm long with one longer and several shorter simple setae, tibia ~0.09 mm long with multiple simple spine-like setae of different lengths, and tarsungulum ~0.05 mm long with two spines laterally opposite to each other.

Abdomen ~1.36 mm long, abdomen units 1–9 wider than long in dorsal view (between 0.05 and 0.18 mm long and between 0.15 and 0.3 mm wide), segment 5 widest, trunk end narrowest (Figure 10A,H). Each unit laterally on both sides with several setae. Trunk end with two urogomphi posteriorly (urogomphi ~0.18 mm long). Urogomphi with two parts: proximal part wider, tapering posteriorly, with several setae laterally, at least one seta fringed (Figure 10G), distal part thinner, much shorter, with one short seta laterally and long seta posteriorly at functionally posterior tip (Figure 10G,I). Pygopod elongated, cylindric with two simple setae posteriorly, urogomphi longer than trunk end, 1.2× (trunk end ~0.15 mm long; Figure 10G).

### 3.10. Results of the Shape Analysis

The shape analysis of 580 specimens (analysis 2 without the strongly polarising specimens, see Material and Methods) resulted in six effective Principal Components (PCs) (Supplementary Files S08 and S09).

PC1 explains 49.53% of the total variance. It is mostly influenced by the length of the head capsule and mandibles. High values indicate a shorter head capsule and longer, more prominent mandibles. Low values indicate a longer head capsule with more protruding labrum or nasale and shorter, less-curved mandibles.

PC2 explains 24.19% of the total variance. It is mostly influenced by the width of the head capsule, either posteriorly or anteriorly, and the width of mandibles, either proximally or distally at the functionally anterior tip. High values indicate a narrower posterior part of the head capsule compared to the rest of the head and mandibles that are wide proximally but very pointed at the tip. Low values indicate a head capsule of more or less continuous width and mandibles of also quite continuous width.

PC3 explains 9.32% of the total variance. It is mostly influenced by the shape of the mandibles. High values indicate a proximally wider mandible with a blunt tip. Low values indicate a proximally slimmer mandible with a pointed tip.

PC4 explains 4.37% of the total variance. It is mostly influenced by the length of the head capsule and the curvature of the mandibles. High values indicate a shorter head capsule and rather straight mandibles. Low values indicate a longer head capsule and more curved mandibles.

PC5 explains 3.11% of the total variance. It is mostly influenced by the shape of the antero-median edge of the head capsule and the length of the mandibles. High values indicate a rather straight anterior edge of the head capsule and relatively short mandibles. Low values indicate a more pronounced antero-median protrusion of the head capsule and relatively long mandibles.

PC6 explains 1.93% of the total variance. It is mostly influenced by the shape of the head capsule and the curvature of the mandible. High values indicate a more oval head capsule with less curved mandibles. Low values indicate a more rectangular head capsule with more curved mandibles.

**Figure 11:**
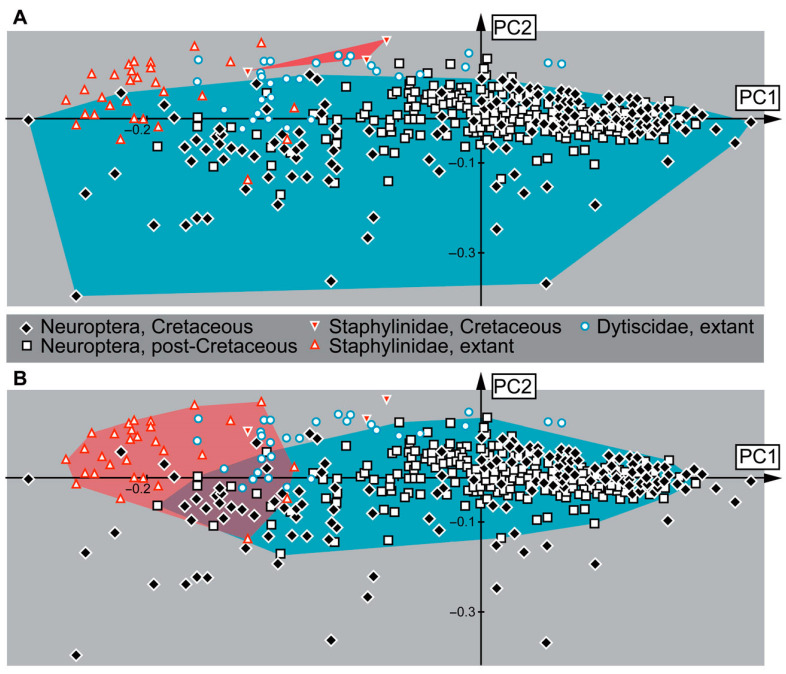
Scatter plot of principal component 2 (PC2) over principal component 1 (PC1) of the outline analysis of head capsules with mandibles of fossil and extant predatory insect larvae; the colour-marked areas represent the occupied morphospaces of rove beetle larvae (red) and lacewing larvae (blue). (**A**) Occupied morphospaces colour-marked for the Cretaceous. (**B**) Occupied morphospaces colour-marked for the extant fauna.

When plotting principal components 1 and 2, the areas occupied by extant and fossil larvae of all studied groups (Staphylinidae, Dytiscidae, Neuroptera) overlap in the morphospace (Figure 11). However, there are certain patterns detectable. For example, the dominance of specimens of Neuroptera is obvious; this is likely partially influenced by the large sample size and inclusion of all available ingroups under a single group name. The larvae of Neuroptera plot throughout the whole scatter plot, and they are situated in all four quadrants. However, the specimens plot especially densely around the origin of the plot and on the right half of the plot, both above and below the x-axis. The only other two specimens plotting on the right side of the plot are larvae of Dytiscidae. In the larvae of Neuroptera, we can also observe some extreme morphologies in the specimens with rather low PC2 values. Most specimens of Staphylinidae and Dytiscidae plot in the upper left quadrant of the plot. The larvae of Dytiscidae show more overlap with the larvae of Neuroptera than the larvae of Staphylinidae. Since the extant larvae of Staphylinidae plot more to the left at lower PC1 values than the larvae of Dytiscidae, we can conclude that the larvae of Staphylinidae have longer heads with a more prominent anterior part of the head capsule (labrum/nasale) and shorter, less-curved mandibles than most larvae of Dytiscidae and Neuroptera. Interestingly, two of the three fossil larvae of Staphylinidae plot outside of the morphospace occupancy of extant larvae of Staphylinidae. The two specimens plot above most larvae of Dytiscidae, relatively high on the y-axis but closer to the 0 value on the x-axis. This position indicates that the larvae had shorter head capsules and shorter mandibles with a rather pointed tip in comparison to the extant larvae of Staphylinidae.

## 4. Discussion

### 4.1. General Identity of the Specimens: Rove Beetle Larvae

The overall morphology of the specimens clearly speaks for an ingroup position of Insecta (e.g., three pairs of walking appendages). The absence of wings seems to be an ontogenetic character (indicating the larval state), and the absence of compound eyes indicates that they are larvae of holometabolans. The campodeiform appearance, number of leg elements, continuous tarsus and claw (tarsungulum) and prominent articulated processes on abdomen unit 9 (urogomphi; in most of the specimens) support an interpretation as representatives of Coleoptera and Polyphaga. There are certain similarities also to adephagan diving beetles (as supported by the shape analysis, Figure 11), yet the differences in certain morphological structures, such as the distal parts of the legs, make such an affinity of the fossils unlikely. Overall similarities are high with larvae of Staphylinidae as lain out in the following.

Further identification is more challenging as there is no single feature characterising the larvae of Staphylinidae (see [45] p. 341) and its major ingroups. Many features that characterise most of these larvae can be found convergently in larvae of other beetle groups, and certain ingroups are highly derived, having secondarily lost important apomorphies for the group Staphylinidae. The situation is especially more complicated as some other groups with distinct larvae were recognised as ingroups of Staphylinidae (e.g., Scydmaeninae [4]). Also, the shape analysis with only specimens of Staphylinidae included did not provide a clear differentiation of the different ingroups of Staphylinidae (Supplementary Files S15–S21). Yet we can recognise certain features in the fossils that are known from modern larvae of Staphylinidae.

### 4.2. Identity of Specimen PED 3982

The moulting suture on the head is V-shaped, similar to modern larvae of Staphylinidae (see [45] p. 342). The fossil has three stemmata visible (Figure 1B); although the number of stemmata in the fossil may be higher due to difficult preservation in this area, up to six would be within the known range of extant larvae (see [45] p. 342). A process on the penultimate element of the antenna on the median side is known in extant larvae (see [45] p. 342); such a process seems not visible in the fossil, but this may be due to the unfortunate angles of view.

In modern rove beetle larvae, only a single endite is present on the maxilla (“mala”; see [45] p. 342); this is also the case for the fossil (Figure 1D). The prothorax is larger than the mesothorax in extant larvae (see [45] p. 342) and in the fossil.

Very prominent in the new fossil are the urogomphi on the ninth unit of the abdomen. Here we reach terminological issues (as not unusual in beetle larvae). The structures have been considered cerci by some authors (e.g., [45] p. 342). If these structures are indeed cerci, then they should arise from abdomen segment 11. In that case, the supposed abdomen segment 9 would in fact be the trunk end, a conglomerate of several undifferentiated segments. The latter aspect seems very likely. The supposed abdomen segment 10 (see [45] p. 342) seems to be nothing more than the differentiated anal region, commonly termed pseudopod (see [45] p. 342). In other groups with a similar structure, this is not necessarily considered a segment and is termed pygopod [199,200,201]. Despite the terminological uncertainties, the arrangement of the structures in the abdomen of the new larvae clearly resembles that of many modern rove beetle larvae.

The larva shares features with numerous extant larvae of rove beetle ingroups. Larvae of Proteininae have three or six stemmata, an antenna with three elements, with the second one being the longest, urogomphus longer than the pygopod and with two elements (see [45] p. 344); all the features are also present in the new fossil. However, in modern larvae of Proteininae, the endite of the maxilla is very long and slender (see [45] fig. 34.241 p. 351) and the distal element in the urogomphus is longer (see [45] p. 344), unlike in the fossil.

In extant larvae of Staphylininae, the urogomphus is also prominent and often longer than the pygopod, and the proximal part of urogomphus is longer than the distal part (see [45] p. 346), as in the fossil. However, extant larvae of Staphylininae differ from the fossil in having at least four stemmata, but this may be preservational, and, more importantly, an articulated endite of the maxilla, which is not the case in the fossil. Additionally, it is not clear whether the antennae of the new fossil have three or four elements; the latter state is also known from the extant larvae.

Extant larvae of Tachyporinae have antennae with three elements, the second being the longest, have no articulation of the endite of the maxilla, a maxilla palp with three elements (see [45] p. 346) and an urogomphus with two elements, the proximal one being the longest (see [202] fig. 35 p. 270). All these features are shared with the fossil (Figure 1). However, extant larvae of Tachyporinae have six stemmata; as pointed out, the lower number in the fossil may be an artefact. Hence, the larva shows most similarities with larvae of Tachyporinae.

However, the monophyly of this group is far from clear (see [9] p. 425; [203]), and larvae of closely related lineages share many features and make it difficult to interpret certain morphologies [2]. The fossil may therefore be closely related to Tachyporinae, or at least be a representative of the “tachyporine group” (term from [2]), but the uncertainties in this region of the phylogenetic tree make further conclusions impossible.

### 4.3. Identity of Specimen PED 0067

The specimen is partly damaged and some structures are concealed by bubbles, yet one very prominent feature is readily visible: the endite of the maxilla (mala) is very elongated and reaches beyond the anterior rim of the head capsule (Figure 2F). This is an identifying feature for the group Proteininae (see [45] p. 344 and fig. 34.241 p. 351). Some other aspects, such as details of labrum, antenna, and mandible, are not reliably accessible. The principal mandible shape is compatible with an interpretation as a larva of Proteininae, but processes (see [45] fig. 34.228 p. 350) are not apparent, which might be explained by preservation (or simply concealed by the maxilla). The palp of the maxilla has three elements (Figure 2B), as in extant larvae of Proteininae (see [45] p. 344). Details of the labium are not apparent. The urogomphus is longer than the pygopod as in extant larvae of Proteininae (see [45] p. 344), but the two elements have a similar length (Figure 2A–C), while in extant larvae the distal one should be longer (see [45] fig. 34.276 p. 352). Still, the other aspects, and especially the elongated maxilla, support an interpretation of the fossil as a representative of Proteininae.

### 4.4. Identity of Specimen PED 2115

For this specimen, many details are not available. Yet the available details, including an antenna with three elements (see [45] p. 344) and an urogomphus with a single element (Figure 3A) that is longer than the pygopod (see [45] p. 344), are compatible with an interpretation of the larva as a representative of Omaliinae [204]. One might argue that the protruding structures of the supposed labium are in fact very elongate endites of the maxillae (for comparison see specimen above in Figure 2 and this fossil in Figure 3), indicating a possible relationship to Proteininae; however, the position of the labial palps in the lateral view and other observed details (number of elements of antenna and urogomphus) do not support such an interpretation. Additionally, a small protrusion between the labial palps and maxillary palps possibly represents an endite of the maxilla in lateral view (Figure 3A). Therefore, we suggest this fossil is a larva of Omaliinae.

### 4.5. Identity of Specimen PED 3368

The overall appearance of this specimen already indicates that the larva is a representative of either Paederinae or Staphylininae. Paederinae is a large ingroup of Staphylinidae with about 7600 formally described species. With slightly more than 50 species known by larval stages, less than 1% of the larvae are known [62], and the situation can only be described as poor [63]. Even though Staphylininae is one of the three largest ingroups of Staphylinidae, representatives of Staphylininae have been very poorly sampled [205], similar to the situation with Paederinae.

Modern larvae of Paederinae and Staphylininae (for example, extant larva of *Dinothenarus*) have several large teeth on the anterior margin of the head capsule (see [45] p. 346), which are apparent in the fossil (Figure 4A–C); an additional pair of smaller teeth cannot be resolved in the fossil, but is potentially present. Stemmata (six in modern forms, see [45] p. 346) are not apparent in the fossil (problematic preservation of stemmata appears to be a common theme). Antenna and maxilla are incomplete and offer no reliable characters. The mandible is well preserved (Figure 4B) and resembles that of many modern-day larvae of Paederine and Staphylininae, but is overall character-poor, not providing a striking feature. The labium appears to have two elements (Figure 4B), the proximal one being longer, as in extant larvae of Paederinae and Staphylininae (see [45] p. 346). However, the tibia of the fossil is not as elongated as in Paederinae (see [45] p. 346). In addition, at the distal part, the tibia bears rows of short setae representing a tibial comb (Figure 4D), also known from the extant representatives of Staphylininae. The tarsungulum of the fossil bears two small setae and a third minute seta proximally (Figure 4E). This is another feature known from the older instars of Staphylininae. Considering that the specimen is damaged and obscured by the other syninclusions and that features such as urogomphi and pygopod are not discernible, we refrain from identifying this larva deeper than a larva of a group including both Paederinae and Staphylininae. Interestingly, in the shape analysis, the specimen did not plot close to modern representatives of the group (Figure 11).

### 4.6. Identity of Specimens in PED 3744

Many details of the three specimens are not available or are not well preserved. For example, even the number of elements in the antenna and urogomphus is not very apparent. The maxilla with a moderately expressed endite and a palp with three elements may be compatible with numerous ingroups of Staphylinidae. Despite this uncertainty concerning the relationships of these larvae, the piece adds important information: the abundance of rove beetle larvae in the Kachin amber forest was high enough to preserve three larvae together in a single piece of amber.

### 4.7. Identity of the NIGPAS Specimen

Only a few features are visible in this specimen, as some details are obscured by impurities in the amber. The antenna appears to have four elements, consistent with characteristics of Steninae, Staphylininae, or Paederinae. A strongly forward-protruding labium, as seen in this fossil (Figure 7D), is also typical of larvae in these groups. The urogomphus has two elements and appears short, possibly shorter than the pygopod, though the exact length is unclear due to the impurities of the amber and the viewing angle, which obscure the pygopod end. Similar antenna, labium, urogomphi, and pygopod structures are found in larvae of *Philonthus* (see [206] figs. 78, 80), or similar legs (see [207] fig. 25). Based on these features, we suggest that this specimen is a fossil larva of Staphylininae.

### 4.8. Identity of Specimens in PED 1302

The adult individuals (besides a smaller one) can be identified as rove beetles based on their short elytra. The larvae are also likely larvae of Staphylinidae based on their overall appearance, the presence of urogomphi and a pygopod. Overall the larvae resemble those in amber PED 3744, but also show differences in the penultimate element of antennae and shape of ligula (compare Figure 5C,D and Figure 8E). Although one of the larvae in the piece provides us with some information, many features cannot be seen in detail (i.e., details that are only accessible in dorsal view, or very small details like hairs or part of mouthparts). However, certain features are available: the anterior margin of the head capsule does not seem to have teeth-like structures; the antennae are relatively stout with three elements; the endite on the maxilla (mala?) is long and thin; the labium is relatively short with palps that are shorter than anteriorly tapering ligula, protruding almost to the anterior margin of the head capsule; the elongated thin tibia that tapers distally; the relatively wide and short urogomphi have a single element, and the short pygopod is shorter than the urogomphi. Even though the general appearance of the larva reminds strongly of several larvae represented in figures in Frank (see [45] p. 348) and the described extant larva of *Glypholoma* (see [51] figs. 2–8, 13, 17), all morphological features listed here do not seem to occur together in a single extant larva. There is also a possibility that due to preservation some of the morphological characters are not discernible, which could also lead to a false identification. Nevertheless, this amber piece is of great importance since the syninclusions demonstrate an even higher density of larvae than PED 3744 and, in addition, demonstrate the co-occurrence of larvae and adults.

### 4.9. Identity of Specimen PED 4084

For this specimen, several features are discernible, and we can identify it as a larva of Piestinae. The antennae have three elements, where the penultimate element is the longest and bears a cone-shaped sensory process, similar to that in known extant specimens. The mandibles of the fossil are asymmetrical; one has four teeth, while the other has three teeth. This feature is also known from extant larvae (see [45] fig. 34.230 p. 350). The maxilla is also similar to the maxilla of modern larvae with fixed endite (mala?) and maxillary palp with three elements, of which the penultimate one is longer than the proximal element (see [45] fig. 34.243 p. 351). The urogomphus has two elements, with a much longer proximal part compared to the distal part. Both parts together are also longer than the pygopod. Additionally, one of the longer setae on the proximal part of the urogomphus seems to have a fringed tip (Figure 10G). These characters can also be observed in extant larvae of Piestinae (see [202] fig. 26; see [45] fig. 34.278 p. 352).

### 4.10. Life Habits

Since habitats of modern larvae are manifold and there is phylogenetic uncertainty, conclusions based on modern counterparts are limited to a certain degree. Due to the preservation of the fossils in amber, the original habitat of the fossils might have been in decaying plant material or under the bark of fallen trees, where some modern larvae are known to live (see [45] p. 341). The large, sickle-shaped mandibles suggest that the larvae were predatory.

Some modern predatory larvae of Staphylinidae are known to hunt fly larvae (see [44] p. 22) or other soft larvae. The soft larvae of stratiomyomorphan flies are among the most common larvae in Kachin amber [208,209,210] and may constitute a possible prey for the new fossil.

### 4.11. Faunal Changes over Time

Rove beetles seem very dominant in the modern fauna [6,8]. In general, the success of holometabolans has been attributed to an ecological differentiation between larva and adult [211]. Yet this differentiation seems not strongly expressed in rove beetles, as adults and larvae co-occur in the same habitat (co-occurrence is also demonstrated for the fossils, Figure 8 and Figure 9); both are often predatory (see [44] p. 22) using (mostly) their prominent mandibles.

Due to the shortening of the elytra (exposing the posterior abdomen) in the adults, they even have a certain larva-like appearance; at least it provides them with more flexibility of the posterior abdomen, similar to that in the larvae. This flexibility might have been originally coupled with the ability to follow prey into crevices (see [212] p. 2). Additionally, this flexibility was advantageous in combination with the defensive glands [213], as demonstrated by other animals with posterior defensive glands that also have a highly flexible posterior trunk, such as whip scorpions (Thelyphonidae) [214,215] or ants (Formicidae) [216,217].

In the modern fauna, rove beetles are important predators also of pests (see [45] p. 343) and can be encountered regularly in the field. They seem not to have been so common yet in the Cretaceous, with only few larvae found, although quite some diversification has occurred already, as demonstrated by the quite numerous fossil adults. To provide a perspective, several hundred larvae of lacewings have been reported from Kachin amber [43,80,87,92,114,115,162,163,164,166,167,169,181,218,219,220,221,222,223,224,225,226,227,228], although in the modern fauna the group of lacewings (Neuroptera) has only 10% of the species richness of Staphylinidae [177].

Abundances of rove beetles in general and rove beetle larvae in particular may have increased later, possibly replacing some predators that decreased after the Cretaceous, such as the mentioned lacewing larvae [93,221]. The shape analysis shows that two of the three fossils reported here, which could be included, plot just outside the range of lacewings, indicating a possible ecological differentiation from them in the Cretaceous. Indeed, areas in which extant rove beetle larvae overlap with lacewing larvae are those that were either occupied by Cretaceous fossils (lost in the modern fauna) or are at least less densely populated by extant lacewing larvae (Figure 11). This gives a first indication of which animals substituted the morphologies lost from lacewing larvae since the Cretaceous [221]. The causal relation must remain unclear. We could expect either that rove beetles simply filled up ecological roles no longer present after certain lacewings went extinct, or a competition in which rove beetles succeeded, causing the extinction of certain lacewings.

It is still possible that a sampling bias has led to currently so few fossil rove beetle larvae. However, other lineages of Holometabola, such as Lepidoptera, also show a pattern of earlier diversification [229,230], but only later increased relative abundance [231]. Although the sample size of the shape analysis is still quite small for rove beetles, there is a signal that rove beetle larvae might have replaced some of the lost diversity, and, with this, ecological function, of lacewing larvae.

## Data Availability

The original contributions presented in this study are included in the article/Supplementary Materials. Further inquiries can be directed to the corresponding author. Figure 1, Figure 2, Figure 3, Figure 4, Figure 5, Figure 6, Figure 7, Figure 8, Figure 9 and Figure 10 in high resolution are available at https://doi.org/10.5281/zenodo.15450407 (accessed on 17 May 2025).

## Supplementary Materials

The following supporting information can be downloaded at https://www.mdpi.com/article/10.3390/insects16090910/s1: Supplementary Table S1. Information on the specimens included in the shape analyses; Supplementary Files S01–S21. Results of the shape analyses.
