# Supplementary figures and images for: Possible Fossil Larvae of Staphylinidae from Kachin Amber and a Quantitative Morphological Comparison Indicate That Rove Beetle Larvae Partly Replaced Lacewing Larvae
Source: Insects. 2025 Sep 1;16(9):910. doi: 10.3390/insects16090910 (PMC12470659; doi:10.3390/insects16090910)

-2S.D.

Mean

+2S.D.

PC1

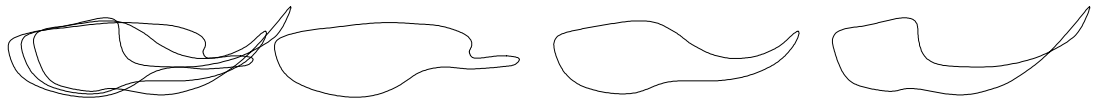

PC2

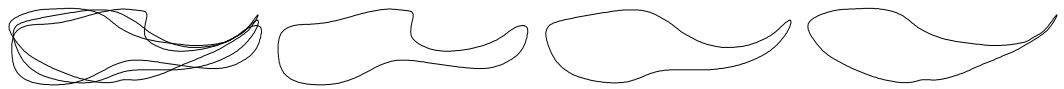

PC3

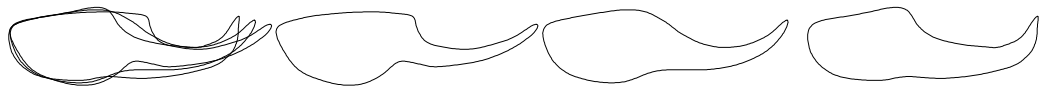

PC4

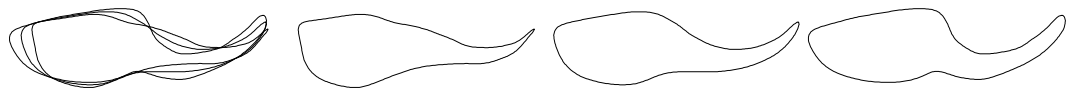

PC5

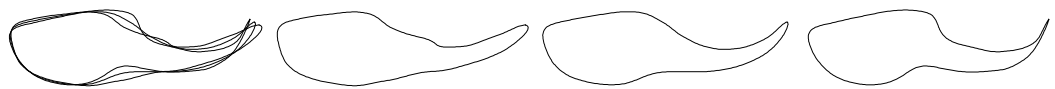

-2S.D.

Mean

+2S.D.

PC6

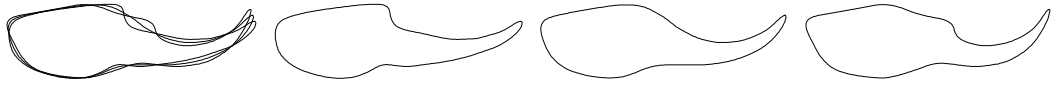

Supplement: Supplementary file 1 [file insects-16-00910-s001.zip › SUPPLEMENT_Staphylinidae/File_S02_all_analysis1_factor_loadings.pdf]

-2S.D.

Mean

+2S.D.

PC1

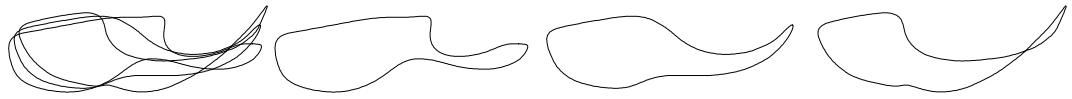

PC2

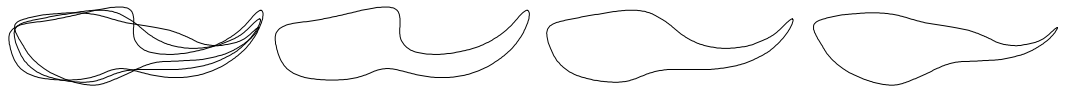

PC3

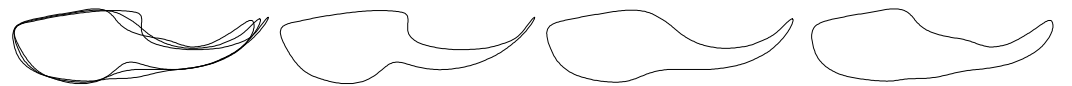

PC4

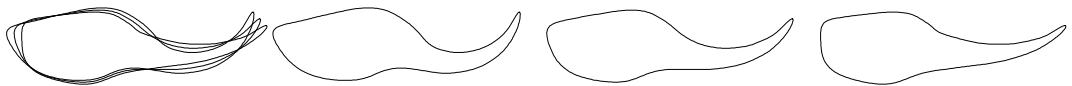

PC5

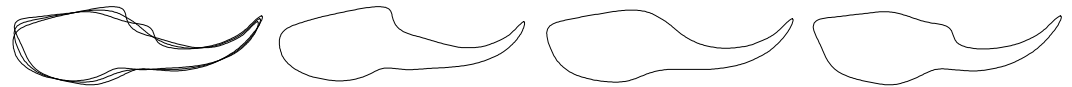

-2S.D.

Mean

+2S.D.

PC6

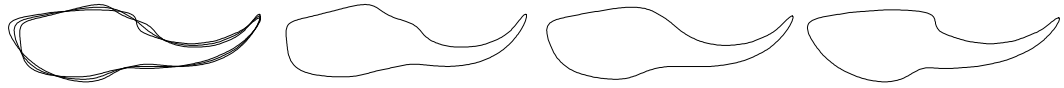

Supplement: Supplementary file 1 [file insects-16-00910-s001.zip › SUPPLEMENT_Staphylinidae/File_S09_excl_pol_analysis2_factor_loadings.pdf]

-2S.D.

Mean

+2S.D.

PC1

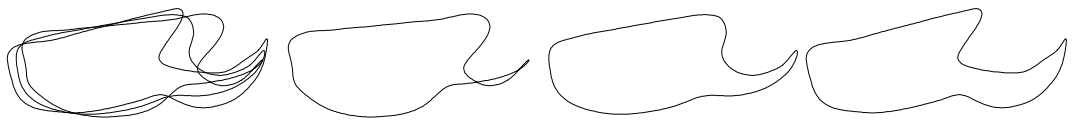

PC2

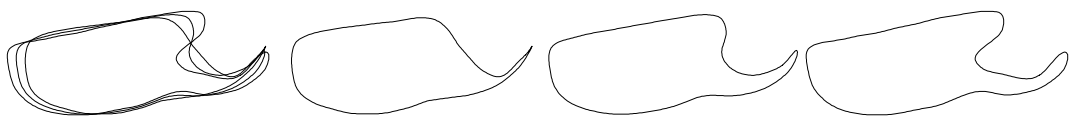

PC3

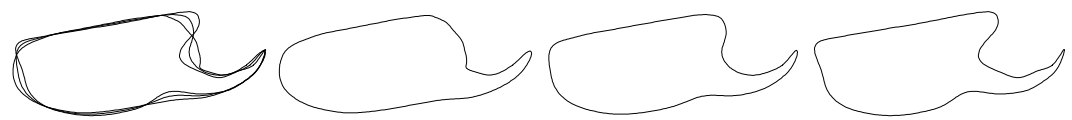

PC4

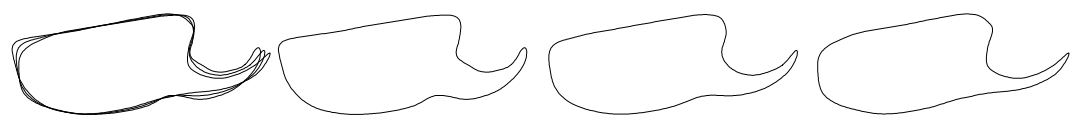

PC5

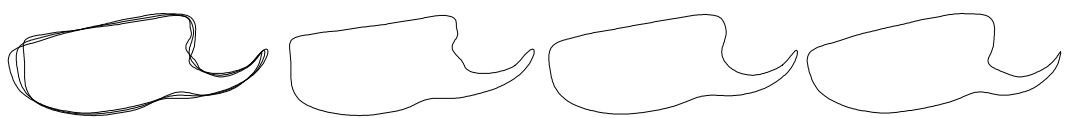

Supplement: Supplementary file 1 [file insects-16-00910-s001.zip › SUPPLEMENT_Staphylinidae/File_S16_only_Staphyl_analysis3_factor_loadings.pdf]
